# Impact of Vortioxetine on Synaptic Integration in Prefrontal-Subcortical Circuits: Comparisons with Escitalopram

**DOI:** 10.3389/fphar.2017.00764

**Published:** 2017-10-26

**Authors:** Shreaya Chakroborty, Thomas R. Geisbush, Elena Dale, Alan L. Pehrson, Connie Sánchez, Anthony R. West

**Affiliations:** ^1^Department of Neuroscience, Chicago Medical School, Rosalind Franklin University of Medicine and Science, North Chicago, IL, United States; ^2^Department of Neuroscience, Novartis Institutes for BioMedical Research, Cambridge, MA, United States; ^3^Department of Psychology, Montclair State University, Montclair, NJ, United States; ^4^Department of Clinical Medicine, Aarhus University, Aarhus, Denmark

**Keywords:** vortioxetine, escitalopram, serotonin, prefrontal cortex, nucleus accumbens, lateral septum, cingulate cortex

## Abstract

Prefrontal-subcortical circuits support executive functions which often become dysfunctional in psychiatric disorders. Vortioxetine is a multimodal antidepressant that is currently used in the clinic to treat major depressive disorder. Mechanisms of action of vortioxetine include serotonin (5-HT) transporter blockade, 5-HT_1A_ receptor agonism, 5-HT_1B_ receptor partial agonism, and 5-HT_1D_, 5-HT_3_, and 5-HT_7_ receptor antagonism. Vortioxetine facilitates 5-HT transmission in the medial prefrontal cortex (mPFC), however, the impact of this compound on related prefrontal-subcortical circuits is less clear. Thus, the current study examined the impact of systemic vortioxetine administration (0.8 mg/kg, i.v.) on spontaneous spiking and spikes evoked by electrical stimulation of the mPFC in the anterior cingulate cortex (ACC), medial shell of the nucleus accumbens (msNAc), and lateral septal nucleus (LSN) in urethane-anesthetized rats. We also examined whether vortioxetine modulated afferent drive in the msNAc from hippocampal fimbria (HF) inputs. Similar studies were performed using the selective 5-HT reuptake inhibitor [selective serotonin reuptake inhibitors (SSRI)] escitalopram (1.6 mg/kg, i.v.) to enable comparisons between the multimodal actions of vortioxetine and SSRI-mediated effects. No significant differences in spontaneous activity were observed in the ACC, msNAc, and LSN across treatment groups. No significant impact of treatment on mPFC-evoked responses was observed in the ACC. In contrast, vortioxetine decreased mPFC-evoked activity recorded in the msNAc as compared to parallel studies in control and escitalopram treated groups. Thus, vortioxetine may reduce mPFC-msNAc afferent drive via a mechanism that, in addition to an SSRI-like effect, requires 5-HT receptor modulation. Recordings in the LSN revealed a significant increase in mPFC-evoked activity following escitalopram administration as compared to control and vortioxetine treated groups, indicating that complex modulation of 5-HT receptors by vortioxetine may offset SSRI-like effects in this region. Lastly, neurons in the msNAc were more responsive to stimulation of the HF following both vortioxetine and escitalopram administration, indicating that elevation of 5-HT tone and 5-HT receptor modulation may facilitate excitatory hippocampal synaptic drive in this region. The above findings point to complex 5-HT receptor-dependent effects of vortioxetine which may contribute to its unique impact on the function of prefrontal-subcortical circuits and the development of novel strategies for treating mood disorders.

## Introduction

Prefrontal-subcortical circuits play an important role in mediating motor function, motivated behaviors, attention, associative learning, and emotional processes (Mogenson et al., 1980; Floresco, 2015). Modulation of glutamatergic and GABAergic transmission by monoaminergic neuromodulators such as serotonin (5-HT) is critical for neural processing in these circuits (Marek and Aghajanian, 1998; Puig and Gulledge, 2011; Celada et al., 2013; Pehrson et al., 2016). Moreover, 5-HT dysfunction in the prefrontal cortex (PFC) and associated limbic regions has been implicated in numerous neuropsychiatric disorders including major depressive disorder (MDD), schizophrenia, anxiety, attention-deficit hyperactivity disorder, and addiction (Artigas, 2013; Grace, 2016; Dalley and Robbins, 2017). Indeed, neuropsychiatric patients exhibit structural and functional alterations in brain regions which receive dense serotonergic innervation from the brainstem raphe nuclei and express high levels of 5-HT receptors, such as the medial PFC (mPFC), anterior cingulate cortex (ACC), ventral hippocampus (vHC), lateral septal nucleus (LSN), and the medial shell of the nucleus accumbens (msNAc) (Sheehan et al., 2004; Marchand and Bennett, 2005; Calhoon and Tye, 2015; Heller, 2016). Current therapeutic strategies targeting 5-HT systems for the treatment of neuropsychiatric disorders include selective serotonin reuptake inhibitors (SSRIs) and 5-HT receptor agonists/antagonists (Artigas, 2013), however, these pharmacological approaches are not always effective, exhibit slow onset of action, and may produce considerable side effects.

The antidepressants vortioxetine and escitalopram are currently used in the clinic for the treatment of MDD. In addition to enhancing serotonergic transmission by blocking the 5-HT transporter (SERT), vortioxetine has effects on multiple 5-HT receptor subtypes (Bang-Andersen et al., 2011; Sanchez et al., 2015; Dale et al., 2016; Pehrson et al., 2016). Vortioxetine is a 5-HT_1A_ agonist, 5-HT_1B_ partial agonist, and an antagonist at the 5-HT_1D_, 5-HT_3_, and 5-HT_7_ receptors (Mørk et al., 2012). In contrast, escitalopram is a highly specific SSRI with little affinity for 5-HT receptors (Owens et al., 2001). An acute dose of vortioxetine has been shown to augment 5-HT release in the vHC to more than twice that observed following escitalopram (Pehrson et al., 2013), suggesting that 5-HT receptor modulation contributes significantly to the facilitatory effect of vortioxetine on serotonergic transmission (Sanchez et al., 2015; Pehrson et al., 2016). Moreover, similar facilitatory effects on 5-HT release were observed in the mPFC and vHP following coadministration of SSRIs and a 5-HT_3_ receptor antagonist, indicating that vortioxetine may potentiate the effects of SERT inhibition through combined antagonism of the 5-HT_3_ receptor (Mørk et al., 2012; Riga et al., 2016). Local 5-HT_3_ receptor antagonism or GABA-B receptor agonism in the vHPC augmented the SSRI effects, thus the potentiation of 5-HT release is likely to be mediated by intrinsic mechanisms (Riga et al., 2016). Interestingly, 5-HT_3_ receptors are exclusively expressed in GABAergic interneurons in the cortex and vHC (Puig et al., 2004; Lee et al., 2010), and activate feed-forward inhibition of pyramidal neurons. Observations that vortioxetine (but not escitalopram) increased the spontaneous firing of pyramidal neurons in the mPFC via a 5-HT_3_ receptor dependent mechanism, suggest that blockade of 5-HT_3_ receptor activation may decrease GABA tone on 5-HT terminals and disinhibit cortical output (Riga et al., 2016). Vortioxetine also functions as an antagonist at 5-HT_1B_ autoreceptors in the HC and NAc, which can result in decreased inhibition of 5-HT release (Artigas, 2013). Together, these multimodal effects of vortioxetine on serotonergic transmission may modulate information transmission from the vHC and mPFC to other functionally coupled cortical and subcortical networks.

Currently, while it is likely that modulation of serotonergic transmission by vortioxetine may alter the feed-forward inhibitory regulation of prefrontal projection neurons and lead to increased output from pyramidal neurons (Sanchez et al., 2015; Riga et al., 2016), the impact of vortioxetine on projection neurons in the ACC, LSN, and msNAc is not known. Given the abundant distribution of SERT and various 5-HT receptors in the above brain regions (Biegon et al., 1982; Pazos et al., 1985; Pehrson and Sanchez, 2014; Pehrson et al., 2016), it is likely that further characterization of the impact of vortioxetine and escitalopram on synaptic transmission and neuron activity in prefrontal-subcortical circuits will reveal novel strategies for treating mood disorders such as MDD. To address this issue, the current study examined the impact of systemic escitalopram and vortioxetine administration on spontaneous neuronal activity and afferent drive elicited by stimulation of the mPFC or hippocampal fimbria (HF) in the ACC, msNAc, and LS in intact rats.

## Materials and Methods

### Subjects

Data are derived from 103 male Sprague Dawley rats (Harlan, Madison, WI, United States) weighing approximately 300–400 g (8–10 weeks of age) at the time of experimentation. Rats were housed two per cage under standard laboratory conditions (21-23°C) and maintained on a 12:12 h light/dark cycle with food and water available *ad libitum*. All animal protocols were approved by the Rosalind Franklin University of Medicine and Science Institutional Animal Care and Use Committee and adhered to the *Guide for the Care and Use of Laboratory Animals* published by the USPHS.

### Drug Treatment

Vortioxetine (0.8 mg/kg) or escitalopram (1.6 mg/kg) was dissolved in a vehicle consisting of 20% 2-Hydroxypropyl-β-cyclodextrin in physiological saline. All compounds were prepared daily and administered intravenously (i.v.) through the lateral tail vein to enable rapid examination of potential acute effects of drug on neuronal activity. Drug doses and route were derived from previous studies (Riga et al., 2016). Projection neuron activity was recorded prior to and up to 3 h following vehicle or drug administration (10 min intervals).

### Surgery

Animals were anesthetized with urethane (1.5 g/kg) and placed in a stereotaxic apparatus. The level of anesthesia was periodically verified via the hind limb compression reflex and maintained using supplemental administration as previously described (Sammut et al., 2010; Padovan-Neto et al., 2015). Temperature was monitored using a rectal probe and maintained at 37°C using a heating pad (Vl-20F, Fintronics Inc., Orange, CT, United States). Burr holes (∼2 mm in diameter) were drilled in the skull overlying regions of interest. The dura mater was resected, and the stimulating and recording electrodes were lowered into the brain using a Narishige micromanipulator. Bipolar stimulating electrodes were implanted ipsilaterally into the mPFC and the HF as previously described (Dec et al., 2014). A bipolar recording electrode was also implanted into the contralateral mPFC for the monitoring of local field potentials (LFPs) (Tseng et al., 2011). Glass extracellular recording electrodes were implanted initially into the ACC and subsequently advanced into the LS and msNAc ipsilateral to mPFC and HF stimulating electrodes. Coordinates for electrode placements are as follows: from bregma: mPFC – anterior: 3.2 mm, lateral: 0.8 mm; fim – posterior: 1.4 mm, lateral: 2 mm; ACC/LS/NAc – anterior: 1.2–1.8 mm, lateral: 0.6–1.4 mm; ventral from the surface of the brain: mPFC: 4.4 mm, HF: 4 mm, ACC: 1.0–2.5 mm, LS: 3.0–5.5 mm, NAc: 5.5–8.0 mm (Paxinos and Watson, 1998).

### Extracellular Recordings and Electrical Stimulation

Recording microelectrodes were manufactured from 2.0 mm OD borosilicate glass capillary tubing and filled with sodium chloride (2M) solution (Ondracek et al., 2008; Threlfell et al., 2009; Sammut et al., 2010; Padovan-Neto et al., 2015). Electrode impendence in situ was 15–25 MΩ. The signal to noise ratio for all recordings was ≥4:1. Electrical stimuli (duration = 500 μs, intensity = 600–1000 μA, in steps of 200 μA) were generated using a Grass stimulator and delivered in single pulses (0.5 Hz) over 50 consecutive trials via the mPFC electrode implanted ipsilateral to the recording pipette. In order to isolate single units, extracellular microelectrodes were lowered incrementally through the ACC, LS, and msNAc using a micromanipulator (MO-8, Narishige) while single pulse electrical stimuli (see above) were administered to the mPFC (Dec et al., 2014). Once a cell was detected, the position of the recording electrode was adjusted to maximize the spike signal to background noise ratio (≥4:1). In a subgroup of neurons/animals, stimulation currents delivered to the HF were titrated to an intensity (range, 200–1300 μA) that reliably evoked spike activity approximately 50% of the time to enable comparisons of evoked activity across vehicle and drug treatment groups (Padovan-Neto et al., 2015). Following isolation of single units using mPFC stimulation, non-evoked basal spike activity and cortical LFPs were recorded for 3 min (Tseng et al., 2011). The response to mPFC stimulation was reaffirmed before searching for the next cell. Typically, 2–4 cells were recorded in as many tracks per animal. Extracellular electrode potentials were acquired and analyzed as previously described (Dec et al., 2014). Neurons exhibiting spike characteristics that could be described as fast-spiking [GABAergic interneurons which respond to low intensity stimulation with a high-frequency train of short duration (<0.9 ms) action potentials] were excluded (Padovan-Neto et al., 2015).

### Data Analysis and Statistics

The influence of vortioxetine or escitalopram on spontaneous and evoked activity of electrophysiologically identified ACC, LS, and msNAc neurons was determined in between-subjects studies as indicated. Firing rate histograms and peri-stimulus time histograms (PSTHs) were constructed (1.0 ms bins) for each recording trial (Dec et al., 2014; Padovan-Neto et al., 2015). Action potential durations were calculated as in Padovan-Neto et al. (2015). Neurons were considered “spontaneously active” if they fired multiple action potentials (>1) during a 3 min recording period. Spike probability was calculated by dividing the number of evoked action potentials (0 or 1 per pulse) by the number of stimuli delivered. Single unit and group data were also summarized using spike latency and standard deviation (SD) of latency plots as indicated. The statistical significance of drug-induced changes in spike activity was determined by using either a Chi-square test or one-way analysis of variance (ANOVA) (Sigma Stat, Jandel). A Tukey *post hoc* test was used to determine which group(s) contributed to overall differences seen with ANOVA.

### Histology

After completion of each experiment, the rat was deeply anesthetized and perfused transcardially with ice-cold saline followed by 10% formalin in buffered phosphate (PB) (EMS, Hatfield, PA, United States). Brains were removed and post-fixed in formalin/sucrose solution (30%) and stored at 4°C until saturated. Brains were then sectioned into 50 μm coronal slices, mounted, and stained with a Neutral Red/Cresyl Violet (10:1) solution to allow for histological assessment of stimulating and recording electrode tracks (Dec et al., 2014; Padovan-Neto et al., 2015).

## Results

### Stimulating and Recording Electrode Placements

All identified cortical stimulating and recording electrode tips were confirmed to lie in the mPFC between 3.1 and 3.3 mm anterior to bregma, 0.2 and 0.8 mm lateral to the midline, and 3.7 and 4.6 mm ventral to the surface of the brain. All identified HF stimulating electrode tips were confirmed to lie between 1.0 and 1.8 mm posterior to bregma, 1.2 and 2.6 mm lateral to the midline, and 3.2 and 4.5 mm ventral to the surface of the brain. Identified placements for extracellular recording electrodes implanted into the ACC were verified to lie between 1.2 and 1.6 mm anterior to bregma, 0.5 and 1.5 mm lateral to the midline, and 0.2 and 3.5 mm ventral to the brain surface. Identified placements for extracellular recording electrodes implanted into the LSN were verified to lie between 1.2 and 1.6 mm anterior to bregma, 0.6 and 1.4 mm lateral to the midline, and 3.0 and 5.5 mm ventral to the brain surface. Identified placements for extracellular recording electrodes implanted into the msNAc were verified to lie between 1.2 and 1.6 mm anterior to bregma, 0.6 and 1.4 mm lateral to the midline, and 5.0 and 7.8 mm ventral to the brain surface. Coordinates are derived from the rat brain atlas of Paxinos and Watson (1998).

### Effects of Acute Antidepressant Treatment on Spontaneous Neuronal Activity in the ACC, LSN, and msNAc

The spontaneous firing activity [firing rate, inter-spike interval (ISI), and coefficient of variability (CV) for the ISI] of ACC, LSN, and msNAc neurons was recorded in vehicle treated controls and rats administered escitalopram or vortioxetine. Firing activity data were grouped according to whether neurons fired spontaneously and were also responsive to mPFC stimulation (mPFC-responsive), and neurons that fired spontaneously but did not respond to mPFC stimulation (non-responsive). No significant differences in firing activity of mPFC-responsive or non-responsive neurons were observed across groups in any of the brain regions examined (**Tables 1–3**; *p* > 0.05, one-way ANOVA). The proportion of spontaneously active cells measured in control and drug treated animals was also similar across groups and brain regions (*p* > 0.05, Chi-square test).

**Table 1:** Summary of spontaneous firing properties of medial prefrontal cortex (mPFC)- and non-mPFC-responsive cells recorded in the anterior cingulate cortex (ACC).

	Spontaneous activity: mPFC-responsive cells			Spontaneous activity: non-mPFC-responsive cells		
	FR (Hz)	ISI (ms)	CV of ISI	FR (Hz)	ISI (ms)	CV of ISI
Control	5.81 ± 1.5	1103 ± 396.0	1.54 ± 0.2	8.87 ± 1.9	424 ± 100.9	1.27 ± 0.1
Vortioxetine	3.49 ± 0.7	683 ± 282.9	1.33 ± 0.1	9.17 ± 2.8	295 ± 87.2	1.15 ± 0.1
Escitalopram	3.63 ± 0.8	981 ± 208.9	1.54 ± 1.0	5.55 ± 0.7	396 ± 86.8	1.12 ± 0.1


*No changes in firing rate, inter-spike interval (ISI), and coefficient of variability (CV) of ISI were observed in the ACC with vortioxetine and escitalopram treatment. Data are presented as Mean ± SEM and analyzed using one-way analysis of variance (ANOVA) with Tukey post hoc analysis (mPFC-responsive: n = 22 cells for control, n = 15 cells for vortioxetine-treated, and n = 26 cells for escitalopram-treated groups; non-mPFC-responsive: n = 39 cells for control, n = 16 cells for vortioxetine-treated, and n = 23 cells for escitalopram-treated groups)*.

**Table 2:** Summary of spontaneous firing properties of mPFC- and non-mPFC-responsive cells recorded in the LS.

	Spontaneous activity: mPFC-responsive cells			Spontaneous activity: non-mPFC-responsive cells		
	FR (Hz)	ISI (ms)	CV of ISI	FR (Hz)	ISI (ms)	CV of ISI
Control	4.10 ± 1.5	1635 ± 632.3	1.73 ± 0.3	12.72 ± 3.0	167 ± 33.9	1.31 ± 0.2
Vortioxetine	3.01 ± 1.3	1278 ± 328.8	1.45 ± 0.2	9.75 ± 3.1	290 ± 73.6	1.33 ± 0.1
Escitalopram	2.91 ± 1.5	2110 ± 719.3	1.12 ± 0.1	4.32 ± 0.7	350 ± 62.9	1.10 ± 0.1


*No changes in firing rate, ISI and CV of ISI were observed in the LS with vortioxetine and escitalopram treatment. Data are presented as Mean ± SEM and analyzed using one-way ANOVA with Tukey post hoc analysis (mPFC-responsive: n = 14 cells for control, n = 13 cells for vortioxetine-treated, and n = 11 cells for escitalopram-treated groups; non-mPFC-responsive: n = 19 cells for control, n = 15 cells for vortioxetine-treated, and n = 15 cells for escitalopram-treated groups)*.

**Table 3:** Summary of spontaneous firing properties of mPFC- and non-mPFC-responsive cells recorded in the medial shell of the nucleus accumbens (msNAc).

	Spontaneous activity: mPFC-responsive cells			Spontaneous activity: non-mPFC-responsive cells		
	FR (Hz)	ISI (ms)	CV of ISI	FR (Hz)	ISI (ms)	CV of ISI
Control	4.26 ± 1.7	1452 ± 714.9	1.42 ± 0.2	13.77 ± 3.2	168 ± 36.9	1.15 ± 0.2
Vortioxetine	2.93 ± 0.8	2235 ± 859.6	1.21 ± 0.2	10.83 ± 2.6	217 ± 96.9	1.29 ± 0.2
Escitalopram	3.03 ± 1.4	1852 ± 624.4	1.54 ± 0.3	12.48 ± 2.2	143 ± 30.1	0.90 ± 0.1


*No changes in firing rate, ISI or CV of ISI were observed with vortioxetine and escitalopram treatment. Data are presented as Mean ± SEM and analyzed using one-way ANOVA with Tukey post hoc analysis (mPFC-responsive: n = 11 cells for control, n = 13 cells for vortioxetine-treated, and n = 12 cells for escitalopram-treated groups ; non-mPFC-responsive: n = 18 cells for control, n = 13 cells for vortioxetine-treated, and n = 19 cells for escitalopram-treated groups)*.

### Effects of Acute Antidepressant Treatment on mPFC-Evoked Neuronal Activity in the ACC, LSN, and msNAc

**FIGURE 1:**
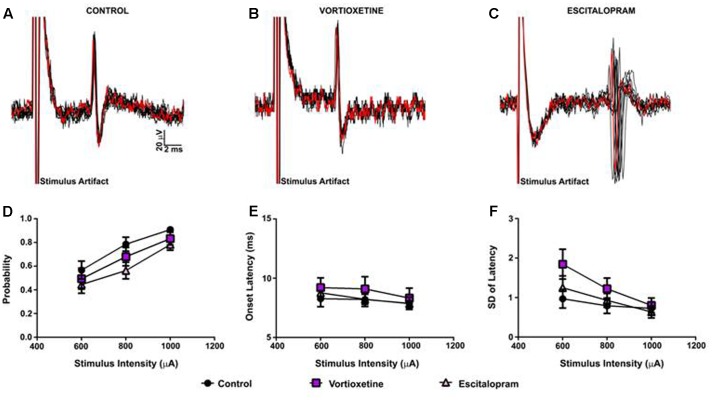
Between groups comparison of the effects of vortioxetine and escitalopram on medial prefrontal cortex (mPFC)-evoked responses recorded from anterior cingulate cortex neurons. **(A–C)** Representative traces of mPFC-evoked responses recorded from isolated anterior cingulate cortex (ACC) neurons (1000 μA stimulus intensity) in controls **(A)**, and following vortioxetine **(B)**, or escitalopram **(C)** administration. Ten consecutive overlaid responses are shown. There was no effect of vortioxetine or escitalopram on **(D)** spike probability, **(E)** onset latency, and **(F)** standard deviation (SD) of latency. Data are presented as Mean ± SEM and analyzed using one-way analysis of variance (ANOVA) (*n* = 32 cells for control, *n* = 21 cells for vortioxetine-treated, and *n* = 31 cells for escitalopram-treated groups).

**FIGURE 2:**
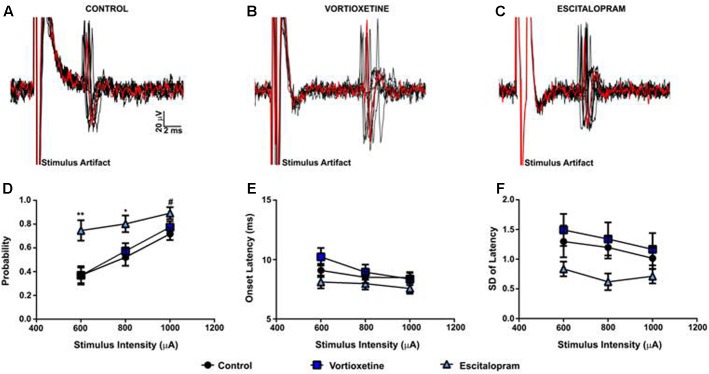
Between groups comparison of the effects of vortioxetine and escitalopram on mPFC-evoked responses recorded from isolated lateral septal nucleus neurons. **(A–C)** Representative traces of mPFC-evoked responses recorded from isolated lateral septal nucleus (LSN) neurons (1000 μA stimulus intensity) in controls **(A)**, and following vortioxetine **(B)**, or escitalopram **(C)** administration. Ten consecutive overlaid responses are shown. **(D)** Escitalopram significantly increased spike probability at 800 μA [*F*(2,60) = 4.165, *p* = 0.0205] and 600 μA [*F*(2,60) = 6.358, *p* = 0.0032] stimulus intensities. There was also a trend toward increased spike probability at 1000 μA [*F*(2,60) = 2.917, *p* = 0.0618] stimulus intensities. *Post hoc* comparisons revealed a significant increase in spike probability following escitalopram administration compared with vortioxetine (^∗∗^*p* < 0.01 at 600 μA, ^∗^*p* < 0.05 at 800 μA) and control groups (^∗∗^*p* < 0.01 at 600 μA, ^∗^*p* < 0.05 at 800 μA, ^#^*p* = 0.06). No changes in onset latency **(E)** and SD of latency **(F)** were observed with either vortioxetine or escitalopram as compared with controls. Data are presented as Mean + SEM and analyzed using one-way ANOVA with Tukey *post hoc* analysis (*n* = 26 cells for control, *n* = 20 cells for vortioxetine-treated, and *n* = 20 cells for escitalopram-treated groups).

**FIGURE 3:**
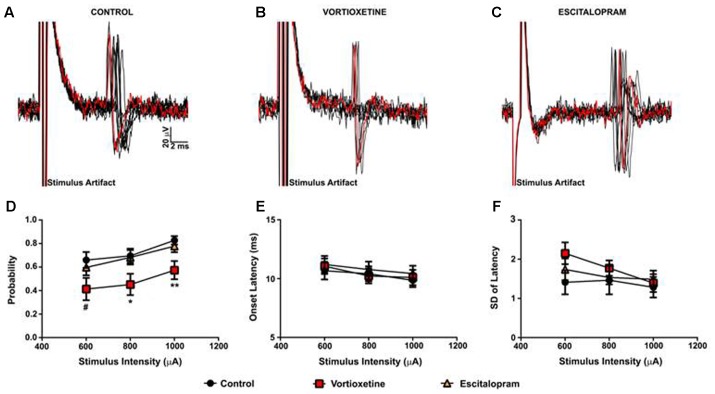
Between groups comparison of the effects of vortioxetine and escitalopram on mPFC-evoked responses recorded from isolated nucleus accumbens shell projection neurons. **(A–C)** Representative traces of mPFC-evoked responses recorded from isolated msNAc neurons (1000 μA stimulus intensity) in controls **(A)**, and following vortioxetine **(B)**, or escitalopram **(C)** administration. Ten consecutive overlaid responses are shown. **(D)** Vortioxetine significantly reduced spike probability at 1000 μA [*F*(2,58) = 5.466, *p* = 0.0067] and 800 μA [*F*(2,58) = 3.627, *p* = 0.0328] stimulus intensities. There was also a trend toward a decrease in spike probability at 600 μA stimulus intensities [*F*(2,58) = 2.653, *p* = 0.0794]. *Post hoc* comparisons revealed a significant decrease in spike probability following vortioxetine administration compared with escitalopram (^∗^*p* < 0.05 at 1000 μA and 800 μA) and control groups (^∗∗^*p* < 0.01 at 1000 μA, ^∗^*p* < 0.05 at 800 μA, ^#^*p* = 0.08). No changes in onset latency **(E)** or SD of latency **(F)** were observed following either vortioxetine or escitalopram administration as compared with controls. Data are presented as Mean ± SEM and analyzed using one-way ANOVA with Tukey *post hoc* analysis (*n* = 21 cells for control, *n* = 20 cells for vortioxetine-treated, and *n* = 20 cells for escitalopram-treated groups).

As described above, in all control and drug treatment groups single units were isolated using low frequency electrical stimulation of the mPFC (**Figures 1A–C**). No significant differences in spike probability, onset latency and SD of spike latency were observed in the ACC across groups (**Figures 1B–F**; *p* > 0.05, one-way ANOVA). In contrast, administration of escitalopram increased the probability of mPFC-evoked spikes in the LSN particularly at lower stimulus intensities (600 μA) [**Figure 2**; *F*(2,60) = 6.358, *p* = 0.0032, one-way ANOVA], whereas vortioxetine was without effect (*p* > 0.05, one-way ANOVA). No significant differences in the onset latency or SD of latency of mPFC-evoked spikes were observed in the LSN across groups (*p* > 0.05, one-way ANOVA). Recordings of mPFC-evoked spike activity in the msNAc revealed that vortioxetine administration decreased the probability of mPFC-evoked spikes particularly at higher stimulus intensities (1000 μA) [**Figure 3**; *F*(2,58) = 5.466, *p* = 0.0067], whereas escitalopram was without effect (*p* > 0.05, one-way ANOVA). No significant differences in the onset latency or SD of latency of mPFC-evoked spikes were observed in the msNAc across groups (*p* > 0.05, one-way ANOVA).

### Effects of Acute Antidepressant Treatment on HF-Evoked Neuronal Activity in the msNAc

**FIGURE 4:**
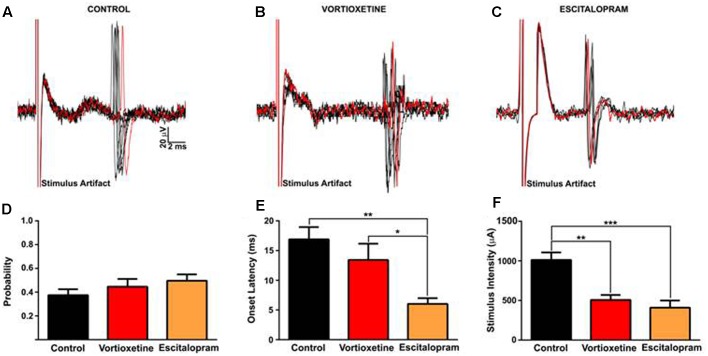
Between groups comparison of fimbria-evoked responses recorded in isolated nucleus accumbens shell projection neurons. **(A–C)** Representative traces of fimbria-evoked responses recorded from isolated msNAc neurons in controls **(A)**, and following vortioxetine **(B)**, or escitalopram **(C)** administration. Ten consecutive overlaid responses are shown. Stimulus intensities were titrated to evoke a 50% response (approximately) to fimbria stimulation, and ranged from 200 to1300 μA **(D)**. **(E)** Escitalopram induced a significant decrease in onset latency of fimbria-evoked responses compared with control and vortioxetine groups [*F*(2,17) = 7.030, *p* = 0.0060]. *Post hoc* comparisons revealed a significant decrease in onset latency following escitalopram administration compared with control (^∗∗^*p* < 0.01) and vortioxetine-treated groups (^∗^*p* < 0.05). **(F)** Vortioxetine and escitalopram significantly decreased the stimulus intensity required to evoke a 50% response [*F*(2,17) = 15.02, *p* = 0.0002]. *Post hoc* comparisons revealed a significant decrease in stimulus intensity post vortioxetine (^∗∗^*p* < 0.01) and escitalopram (^∗∗∗^*p* < 0.001) compared with controls. SD of latency was unchanged following drug treatment (data not shown). Data are presented as Mean ± SEM and analyzed using one-way ANOVA with Tukey *post hoc* analysis (*n* = 8 cells for control, *n* = 6 cells for vortioxetine-treated, and *n* = 6 cells for escitalopram-treated groups).

It is known that antidepressants modulate hippocampal output via serotonergic mechanisms (Sanchez et al., 2015). Because the msNAc also receives dense glutamatergic projections from the vHC (Brog et al., 1993; Groenewegen et al., 1999), we examined the impact of escitalopram and vortioxetine on evoked activity elicited by low frequency electrical stimulation of the HF in a subset of msNAc neurons (**Figure 4**). To control for variance in the placement of stimulating electrodes in the HF fiber tract and potential differences in afferent input to target neurons in the msNAc, in these studies stimulation currents were titrated to an intensity that reliably evoked spike activity approximately 50% of the time and stimulus intensity and onset latency were analyzed as dependent variables. Systemic escitalopram administration induced a significant decrease in the onset latency of fimbria-evoked responses compared with control and vortioxetine groups [**Figure 4E**; *F*(2,17) = 7.030, *p* = 0.0060, one-way ANOVA]. Interestingly, both escitalopram and vortioxetine decreased the current intensity needed to evoke a 50% response to a similar degree [**Figure 4F**; *F*(2,17) = 15.02, *p* = 0.0002, one-way ANOVA], indicating that increased 5-HT tone following SERT inhibition results in an increase in excitatory hippocampal drive to msNAc neurons.

## Discussion

This study examined the impact of acute administration of the SSRI escitalopram and the multimodal antidepressant vortioxetine on spontaneous activity and afferent drive in prefrontal-subcortical circuits in naïve rats. A major finding was that escitalopram administration induced a significant increase in mPFC-evoked spiking in the LSN as compared to vehicle and vortioxetine treated groups. Interestingly, vortioxetine administration decreased mPFC-evoked spiking in the msNAc, whereas escitalopram was without effect. No significant impact of drug treatment on mPFC-evoked responses was observed in the ACC. Both vortioxetine and escitalopram facilitated excitatory hippocampal afferent drive onto msNAc neurons, suggesting that this effect was mediated via increased 5-HT tone and receptor activation induced following SERT inhibition. The observation that escitalopram (but not vortioxetine) decreased the onset latency of fimbria-evoked responses also points to additional complex modulation of 5-HT receptors, and perhaps a non-SERT dependent modulation of hippocampal drive by vortioxetine.

### Escitalopram But Not Vortioxetine Facilitates Prefrontal Activation of LSN Neurons

The mPFC, and in particular, the infralimbic PFC sends a robust projection to the LSN (Sesack et al., 1989; Vertes, 2004; Gabbott et al., 2005). The LSN is also densely innervated by serotonergic afferents from the dorsal raphe nucleus (Köhler et al., 1982; Risold and Swanson, 1997) and expresses numerous 5-HT receptor subtypes (e.g., 5-HT1-4 and 5-HT7) at moderate to high levels (Biegon et al., 1982; Pazos and Palacios, 1985; Pazos et al., 1985; du Jardin et al., 2014). Electrical stimulation of the raphe has been shown to increase the activity of LSN neurons, an effect that is potentiated by antidepressants (Contreras et al., 1989, 1993, 2001; Sheehan et al., 2004). Bath applied 5-HT has complex effects on the membrane activity of septal neurons, but may increase their responsiveness to excitatory drive by suppressing action potential afterhyperpolarization, facilitating the afterdepolarization, and decreasing inhibitory IPSPs (Joëls et al., 1986; Joëls and Gallagher, 1988). More recent studies performed *in vitro* have shown that 5-HT_2A_ receptor stimulation facilitates excitatory synaptic transmission via a presynaptic mechanism (Hasuo et al., 2002).

The above studies are consistent with our observations that escitalopram administration facilitated excitatory afferent drive from the mPFC to LSN neurons as compared to vehicle treated rats. Unexpectedly, vortioxetine did not alter LSN neuron activity evoked by electrical stimulation of the mPFC. One possible explanation for this observation is that vortioxetine acts as a potent 5-HT_1A_ receptor agonist (Bang-Andersen et al., 2011), an effect that would be expected to strongly hyperpolarize LSN neurons (Yamada et al., 2000, 2001) and attenuate the excitatory effects of 5-HT_2A_ receptor activation (see above) induced following SERT inhibition. Vortioxetine is also a 5-HT_1B_ partial agonist and an antagonist at the 5-HT_1D_, 5-HT_3_, and 5-HT_7_ receptors (Mørk et al., 2012), thus actions at these receptor subtypes may also contribute to suppress the apparent facilitatory effects of SERT inhibition on LSN neuron activity. It is unlikely that the lack of effect of vortioxetine on the responsiveness of LSN neurons to mPFC inputs is related to insufficient drug exposure or target occupancy as the current dose of vortioxetine (0.8 mg/kg, i.v.) has been shown to effectively target SERT, 5-HT_1A_, 5-HT_1B_, 5-HT_1D_, 5-HT_3_, and 5-HT_7_ receptors in most of the areas of interest to the current study (Pehrson et al., 2013; Sanchez et al., 2015). Based on binding affinities of vortioxetine and target expression levels in the LSN (Pazos and Palacios, 1985; Pazos et al., 1985; Bang-Andersen et al., 2011; Mørk et al., 2012), the recruitment of functionally relevant 5-HT targets would be expected to approximate the following sequence: 5-HT_3_ > SERT > 5-HT_1D_ > 5-HT_1B_ > 5-HT_1A_ > 5-HT_7_ (Sanchez et al., 2015). Additional studies aimed at determining dose-response relationships and the role of these distinct 5-HT receptor subtypes in mediating the modulatory influence of vortioxetine on the synaptic activation of LSN neurons are needed to resolve this issue.

### Vortioxetine But Not Escitalopram Decreases Prefrontal Activation of msNAc Neurons

Acute vortioxetine administration was found to decrease mPFC-evoked spiking in the msNAc, an effect which was not reproduced by escitalopram. This effect was unexpected given recent studies showing that vortioxetine (using the current dose of 0.8 mg/kg, i.v.) strongly augments mPFC pyramidal neuron firing by antagonizing 5-HT_3_ receptors on GABAergic interneurons and presumably, attenuating feed-forward inhibition of mPFC output (Riga et al., 2016). These studies by Riga et al. (2016) focused on mPFC projection neurons targeting the midbrain, so it is possible that vortioxetine has differential effects on corticoaccumbens versus midbrain projecting mPFC pyramidal neurons. Given that the vast majority of *in vivo* studies indicate that 5-HT has primarily inhibitory effects on striatal projection neurons, a more plausible explanation is that local effects of vortioxetine in the msNAc such as increased inhibitory GABAergic and/or cholinergic drive are responsible for the decrease in corticoaccumbens synaptic activation (reviewed in Pehrson et al., 2016).

In support of the above tenent, *in vitro* electrophysiological studies on the effects of 5-HT on identified striatal fast-spiking, parvalbumin expressing GABAergic interneurons (FSIs) and cholinergic interneurons (ChATs) suggests that 5-HT has an excitatory effect on these cell types largely via activation of 5-HT2C receptor subtypes (Blomeley and Bracci, 2005, 2009; Bonsi et al., 2007). Indeed, striatal FSIs interneurons in particular have extensive inhibitory axo-somatic synapses on numerous projection neurons, which powerfully suppress the activation of these cells by excitatory inputs (Tepper et al., 2004; Mallet et al., 2005). FSIs also exhibit relatively high membrane resistance and are more responsive to cortical drive than striatal projection neurons (Tepper et al., 2004; Mallet et al., 2005; Sharott et al., 2012). Given this, an increase in mPFC pyramidal cell output following vortioxetine administration (Riga et al., 2016) would be expected to preferentially activate msNAc FSIs and suppress projection neuron output. In support of this, we have recently shown that robust burst stimulation (10, 20, and 40Hz) of the frontal cortex produces a persistent GABA-mediated inhibition of local striatal field potentials which is strongest at gamma frequencies that primarily drive FSIs (Jayasinghe et al., 2017). This GABAergic suppression of synaptic activation of striatal neurons by FSIs has also been reported in the NAc during burst stimulation of the mPFC (Gruber et al., 2009). Using *in vivo* intracellular recordings Gruber et al. (2009) showed that while the majority of NAc projection neurons are depolarized by burst stimulation of the mPFC, action potential generation is suppressed in these neurons by a GABA-A receptor dependent mechanism. Thus, robust activation of FSIs by mPFC drive in the presence of elevated 5-HT (and 5-HT_3_ receptor blockade in the mPFC) may result in increased feedforward inhibition and decreased msNAc output following vortioxetine administration. Vortioxetine mediated effects on 5-HT_1B_, 5-HT_1D_, and 5-HT_7_ receptors in the msNAc may also play a role in the observed suppression of corticoaccumbens synaptic activation as these receptor subtypes are expressed in low to moderate levels in this region (reviewed in Pehrson et al., 2016).

### Both Escitalopram and Vortioxetine Increase Hippocampal Activation of msNAc Neurons

The msNAc receives a dense glutamatergic input from the vHC by way of the fimbria/fornix (Kelley and Domesick, 1982; Brog et al., 1993; Groenewegen et al., 1999). Previous electrophysiological studies have shown that stimulation glutamatergic hippocampal afferents depolarize the steady state membrane potential of msNAc projection neurons, (O’Donnell and Grace, 1995; Finch, 1996; Goto and O’Donnell, 2002), thus increasing spike probability (reviewed in O’Donnell, 2003; Goto and Grace, 2008). Prior burst stimulation of hippocampal inputs to the NAc can also facilitate subsequent effects of mPFC stimulation on evoked spike activity (Floresco et al., 2001; Dec et al., 2014).

In the present study, we found that both escitalopram and vortioxetine decreased the current intensity of HF stimulation needed to evoke spike activity with approximately 50% probability. Currently, it is not known whether this apparent increase in vHC-msNAc pathway excitability induced following SERT inhibition is a result of changes in vHP excitability and/or modulation of msNAc projection neuron excitability. Interestingly, both escitalopram and vortioxetine have been shown to augment 5-HT release in the vHC (Pehrson et al., 2013), an effect that in the case of vortioxetine (but not escitalopram), is associated with increased synaptic plasticity (i.e., LTP) and vHC output (Dale et al., 2014, 2016). Vortioxetine also elevates extracellular 5-HT levels in the vHC to more than twice that observed following escitalopram (Pehrson et al., 2013), an effect that may be explained by antagonistic action of the former drug on excitatory 5-HT3 receptors and decreased GABAergic feedforward inhibition (Reznic and Staubli, 1997; Dale et al., 2014). This effect of vortioxetine would also be expected to increase vHC output from pyramidal neurons (Reznic and Staubli, 1997; Dale et al., 2014, 2016). The above studies, together with the observation that escitalopram had more robust excitatory effects on HF-evoked responses than vortioxetine (e.g., escitalopram decreased the onset latency of HF-evoked spikes whereas vortioxetine was without effect), indicate that augmentation of vHC-msNAc pathway excitability through local vHC mechanisms may be less important than facilitatory effects of 5-HT in the msNAc or related brain regions.

## Conclusion

It remains unclear precisely how vortioxetine modulates prefrontal afferent drive to suppress corticoaccumbens transmission and whether/how these alterations contribute to the therapeutic efficacy of this antidepressant. Nonetheless, the current findings that vortioxetine and escitalopram exert distinct and opposite modulatory effects in the msNAc and LSN, respectively (i.e., vortioxetine decreased mPFC drive to msNAc neurons whereas escitalopram enhanced mPFC drive to LSN neurons), point to differential effects of these antidepressant drugs on excitatory synaptic transmission and information integration in prefrontal-subcortical structures. A common effect of both compounds was augmentation of hippocampal drive to msNAc neurons, although escitalopram also modulated the timing of action potential discharge evoked by HF stimulation. Interestingly, acute vortioxetine administration has been shown to exhibit considerable efficacy in the majority of behavioral tests designed to assess antidepressant-like actions, including forced swim, novelty-suppressed feeding, open-field, social interaction, and conditioned fear-induced vocalization tests (reviewed in Sanchez et al., 2015). The potential role of the vortioxetine- and escitalopram-induced changes in prefrontal-subcortical synaptic drive in the above behavioral paradigms will need to be assessed in future studies using animal models of MDD.

## Author Contributions

AW, SC, AP, and CS wrote and edited the manuscript. SC and TG performed all experiments. All authors participated in experimental design and made intellectual contributions to this work.

## Conflict of Interest Statement

ED, AP, and CS are former employees of Lundbeck Research United States, Inc. The other authors declare that the research was conducted in the absence of any commercial or financial relationships that could be construed as a potential conflict of interest.
